# Assessment between antiseptic and normal saline for negative pressure wound therapy with instillation and dwell time in diabetic foot infections

**DOI:** 10.1038/s41598-024-58900-3

**Published:** 2024-05-19

**Authors:** Jingchun Zhao, Kai Shi, Nan Zhang, Lei Hong, Jiaao Yu

**Affiliations:** Department of Burn Surgery, the First Hospital of Jilin University, No. 71 Xinmin Street, Changchun, 130021 People’s Republic of China

**Keywords:** Diabetic foot infection, PHMB, Negative pressure wound therapy, Instillation and dwell time, Endocrine system and metabolic diseases, Metabolic disorders, Skin diseases, Trauma

## Abstract

Negative pressure wound therapy with instillation and dwell time (NPWTi-d) is increasingly used for a diverse range of wounds. Meanwhile, the topical wound irrigation solution consisting of polyhexamethylene biguanide and betaine (PHMB-B) has shown efficacy in managing wound infections. However, the effectiveness of this solution as a topical instillation solution for NPWTi-d in patients with diabetic foot infections (DFIs) has not been thoroughly studied. The objective of this retrospective study was to evaluate the impact of using PHMB-B as the instillation solution during NPWTi-d on reducing bioburden and improving clinical outcomes in patients with DFIs. Between January 2017 and December 2022, a series of patients with DFIs received treatment with NPWTi-d, using either PHMB-B or normal saline as the instillation solution. Data collected retrospectively included demographic information, baseline wound characteristics, and treatment outcomes. The study included 61 patients in the PHMB-B group and 73 patients in the normal saline group, all diagnosed with DFIs. In comparison to patients treated with normal saline, patients with PHMB-B exhibited no significant differences in terms of wound bed preparation time (*P* = 0.5034), length of hospital stay (*P* = 0.6783), NPWTi-d application times (*P* = 0.1458), duration of systematic antimicrobial administration (*P* = 0.3567), or overall cost of hospitalization (*P* = 0.6713). The findings of the study suggest that the use of either PHMB-B or normal saline as an instillation solution in NPWTi-d for DFIs shows promise and effectiveness, yet no clinical distinction was observed between the two solutions.

## Introduction

Diabetic foot ulcer (DFU) is a prevalent and dreadful lower-extremity complication that continues to be a significant public health issue due to its high rates of morbidity and mortality, frequently leading to lower limb amputation if not promptly recognized and effectively managed^1^. Actually, more than half of DFU patients develop infections^2,3^, which are the principal pathology responsible for the majority of diabetic foot complications and are a leading cause for hospital admissions among individuals with diabetes^4,5^.

Diabetic foot infections (DFIs) are defined as infections in soft tissue or bone anywhere below the malleoli in a diabetic person^6^. If the infection advances to the underlying bone, then diabetic foot osteomyelitis develops. Numerous independent predisposing factors contributing to the development of DFIs have been identified, including peripheral neuropathy, immunopathy, wounds that penetrate bones, recurrent foot ulcers, a history of lower extremity amputations, a traumatic etiology, the presence of peripheral arterial disease (PAD) in the involved limb, and renal insufficiency^6^. Approximately 20% of moderate or severe DFIs lead to lower extremity amputation at various levels and dramatically increase mortality eventually^7^.

Various organisms, alone or synergistically, can cause DFIs. To make matters worse, a growing body of studies have suggested the presence of biofilms in most DFU wounds, which subsequently impair or deteriorate the healing process and are associated with poor clinical outcomes^8–10^. In fact, biofilm formation is believed to be one of the most important virulence factors in the pathogenesis of DFIs^11^.

Since the development of standard commercial negative pressure wound therapy (NPWT), subsequent research has consistently validated its positive physiological effects, establishing it as a crucial tool in wound care^12^. NPWT involves the application of subatmospheric pressure to the entire wound site through a specialized dressing system. This system comprises four key components: an open-pore foam sponge (typically made of polyurethane or polyvinyl), a semiocclusive adhesive cover, a negative pressure source, and a fluid collection system. NPWT exerts its effectiveness through four primary mechanisms of action: macrodeformation, drainage of fluids, stabilization of the wound environment and microdeformation^13^. In recent studies, there has been a shift in focus from the four main mechanisms of NPWT to the accompanying secondary effects, such as effects on various cells, bacteria, and surgical wounds^14^.

A variety of systems with newer features and functions are available that have helped to broaden its applications, including negative pressure wound therapy with instillation and dwell time (NPWTi-d). These systems typically perform a repeating cycle of fluid instillation using a predetermined volume, followed by a dwell time during which the wound is washed, and then fluid removal and resumption of negative pressure suction^12^. The use of NPWTi-d devices has been shown to promote increased granulation tissue, faster healing times, and decreased hospital length of stay when compared to standard NPWT^15,16^.

Although a variety of instillation solutions can be used in NPWTi-d, considering the growing diabetic population and the need to achieve more successful outcomes and ultimately avoid amputations, an optimal protocol of care for DFIs integrated with an anti-biofilm strategy is particularly critical. In addition, there has been a focus on measures of wound cleansing whereby debris and exudates are gently and continuously removed to prepare the wound bed for wound closure. For this purpose, physiological solutions or specific disinfectants may be used^17^.

Prontosan® wound irrigation solutions (B.Braun Medical AG, Melsungen, Germany) are developed for cleansing, rinsing and moisturizing of acute and chronic skin wounds, and for the prevention of biofilm. In vitro study that determined the activity of a polyhexamethylene biguanide-betaine (PHMB-B) solution against collection strains and multidrug-resistant nosocomial isolates shows that PHMB-B presented bactericidal activity against all multidrug-resistant clinical isolates tested, including high-risk clones, at significantly lower concentrations and time of activity^18^. Numerous clinical studies have also demonstrated the significantly higher efficacy of PHMB-B irrigation solution in terms of tissue compatibility, fast-acting broad spectrum antimicrobial activity, and its benefits for cleaning, moistening and decontaminating the wound bed, reducing inflammatory signs and antimicrobial usage, as well as accelerating the healing of chronic wounds^19–23^. A systematic review assessed characteristics of various antiseptics and recommended that PHMB-B may be considered as the first-choice agent for infected chronic wounds^24^.

However, the effect of NPWTi-d with PHMB-B in the management of DFIs wounds has not been well determined. Given the high failure rate of DFI treatment^25^, and based on the results of the available studies, we hypothesize that the combination of NPWTi-d with anti-biofilm solution PHMB-B has a synergistic effect by enhancing efficacy in the management of DFIs where biofilms present a continuous challenge to effective microbial control and showing advantages in improving the clinical outcomes of the patients.

The purpose of this case study was to evaluate the clinical outcomes of DFI patients treated with NPWTi-d with PHMB-B irrigation solution (Prontosan® Wound Irrigation Solution).

## Methods

This retrospective, historical, controlled study compared the efficacy of Prontosan® Wound Irrigation Solution with normal saline (NS) as a topical instillation solution during NPWTi-d in the treatment of DFIs.

This study was conducted at a regional care center specializing in burn injuries and wound repair in Changchun, Jilin Province, with approval from the institution’s Ethical Committee. Informed consent for the academic use of medical records was obtained from patients and their legal guardians who participated in the study during hospitalization. All methods in this research was performed in accordance with relevant guidelines and regulations. Confidentiality was maintained all through the study.

### Study populations

A retrospective assessment was conducted on a series of consecutive patients who received treatment and underwent reconstructive surgery for DFIs between April 2018 and December 2020.

Patients who met the inclusion criteria of this study were as follows: (1) Patients aged 18 years or older with a definitive diagnosis of diabetes mellitus and DFU; (2) Diagnosis of DFIs was defined by clinical signs or symptoms of infection (erythema, warmth, tenderness, pain, induration), presence of purulent secretion, elevated white blood cell count, positive culture results from deep wound tissue samples, and/or radiographic evidence of infection; (3) Wound bed preparation was conducted using either Prontosan® Wound Irrigation Solution or NS as the topical wound irrigation solution in the treatment of NPWTi-d. (5) Physical examination techniques such as palpation of pedal pulses or measurement of ankle-brachial index, Doppler ultrasound, and angiography were employed to identify PAD in the affected limbs. NPWTi-d was exclusively administered to patients without apparent vascular compromise as a contraindication; (6) All pertinent data was thoroughly documented and gathered.

The exclusion criteria for patients in both groups included terminal illness, malignancy in the wound, standard negative pressure wound therapy without instillation and dwell time, and incomplete information.

### Study products

The Prontosan® Wound Irrigation Solution, as specified by the manufacturer, consists of 0.1% PHMB, 0.1% undecylenamidopropyl betaine, and 99.8% purified water. PHMB is recognized as an effective and rapid-acting broad-spectrum antimicrobial agent, while betaine serves as a surfactant capable of penetrating the skin to disrupt and eliminate biofilm, wound debris, and slough.

The synergistic action of two essential components provides a suitable option for the efficient cleansing, moistening, and decontamination of wounds, particularly in the context of preventing and eliminating biofilms in chronic wounds. The irrigation solution is conveniently packaged in 350 ml bottles and 40 ml ampoules, and is administered directly from the squeeze bottle. In this study, the 350 ml bottle of Prontosan® Wound Irrigation Solution was utilized.

Wound negative pressure wound therapy (NPWT) was administered using either the polyurethane foam-based RENASYS-F Foam Dressing Kit with Soft Port (Smith & Nephew Medical Ltd.) or the V.A.C.® Therapy System (KCI, an Acelity Company, San Antonio, TX, USA). A customized NPWTi-d protocol was devised as a substitute treatment for the surgical wound immediately following the initial debridement, drawing upon existing literature on NPWTi-d.

### Study protocol

The study compared the clinical outcomes of patients with DFIs treated with NPWTi-d using either PHMB-B or NS. Matching was achieved in terms of wound characteristics such as location, size, and purulence. Wound dimensions (width, length, and depth) were measured upon admission or during surgery. Additionally, demographic data was collected and the overall patient condition was assessed through clinical diagnoses documented in medical records, including evaluation of comorbidities such as obesity, body mass index, and dyslipidemia, glycemic status and hemoglobin A1c, heart and peripheral vascular diseases, pulmonary disease, hepatic dysfunction, renal insufficiency, and smoking status.

Following assessment, a thorough excisional debridement of necrotic and devitalized tissues was conducted. Subsequently, a NPWTi-d system utilizing either PHMB-B or NS was applied during the initial operative visit immediately post-debridement in the operating room. Specifically, the foam sponge was tailored to conform to the wound shape post-debridement. A tube with a soft port at the end was utilized for suction, while another tube with perforations on each side was inserted into the sponge for instillation solution delivery. The distal end of the suction tube was affixed to the central negative pressure system on the wall, while the distal end of the solution delivery tube was linked to the bag containing either PHMB-B or NS solution.

NPWTi-d was administered using either PHMB-B or NS solution to instill and saturate the sponge. This was followed by a dwell time of 10–15 min, and then a continuous negative pressure suction of -125 mm Hg over a 24-h period, with four cycles per day. The dwell time referred to the period in which the solution remained in the foam/wound interface without negative pressure suction. The NPWTi-d system was changed every 4–6 days.

During the surgical procedure, deep wound culture specimens and biopsies were collected. Following admission, all patients received empirical systemic antibiotics, which were modified according to the findings of bacterial cultures and biopsies. The presence of nonviable tissue and the formation of granulation tissue were evaluated in both patients and wounds at each NPWTi-d system change. Wounds that did not show improvement or continued to exhibit culture growth underwent further debridement until infection was eradicated in both patient groups.

The study's endpoint for both groups included determining readiness for wound closure via split-thickness skin graft, consideration of limb amputation due to life-threatening infection or lack of limb salvage options, or the patient was discharged from the hospital upon clearance of infection. The criteria for split-thickness skin graft closure were consistent across both groups: (1) a minimum of two sets of negative culture results and evaluated by an experienced surgeon; (2) presence of healthy granulation tissue formation in the wound bed; (3) absence of nonviable tissue; and (4) no vital tissue exposure in the wound bed (tendon, bone or cartilage).

### Data collection and analysis

Data was gathered from electronic medical records for patients who met the predefined inclusion criteria. The collected data for each patient included demographic information (such as age and sex), baseline wound characteristics, duration of wound bed preparation prior to definitive surgeries, time taken to achieve final healing (in days), length of hospital stay (in days), mean NPWTi-d application times, duration of systematic antimicrobial administration (in days), cost (in CNY), and any existing complications.

The study's findings were presented in numerical and percentage form for categorical variables, and as means ± standard deviation (SD) for continuous variables. Data analysis was conducted using SPSS version 27.0 for Windows (SPSS, Chicago, IL, USA). Student's *t*-test and Chi-square test or Fisher exact test were utilized for assessing between-group disparities in quantitative or qualitative data, as deemed suitable. Statistical significance was defined as a *p*-value less than 0.05.

### Ethics approval and consent to participate

This study was approved by the Institutional Review Board of the First Hospital of Jilin University. Written informed consent was obtained from each participating patient or their legal representatives.

## Results

**Figure 1:**
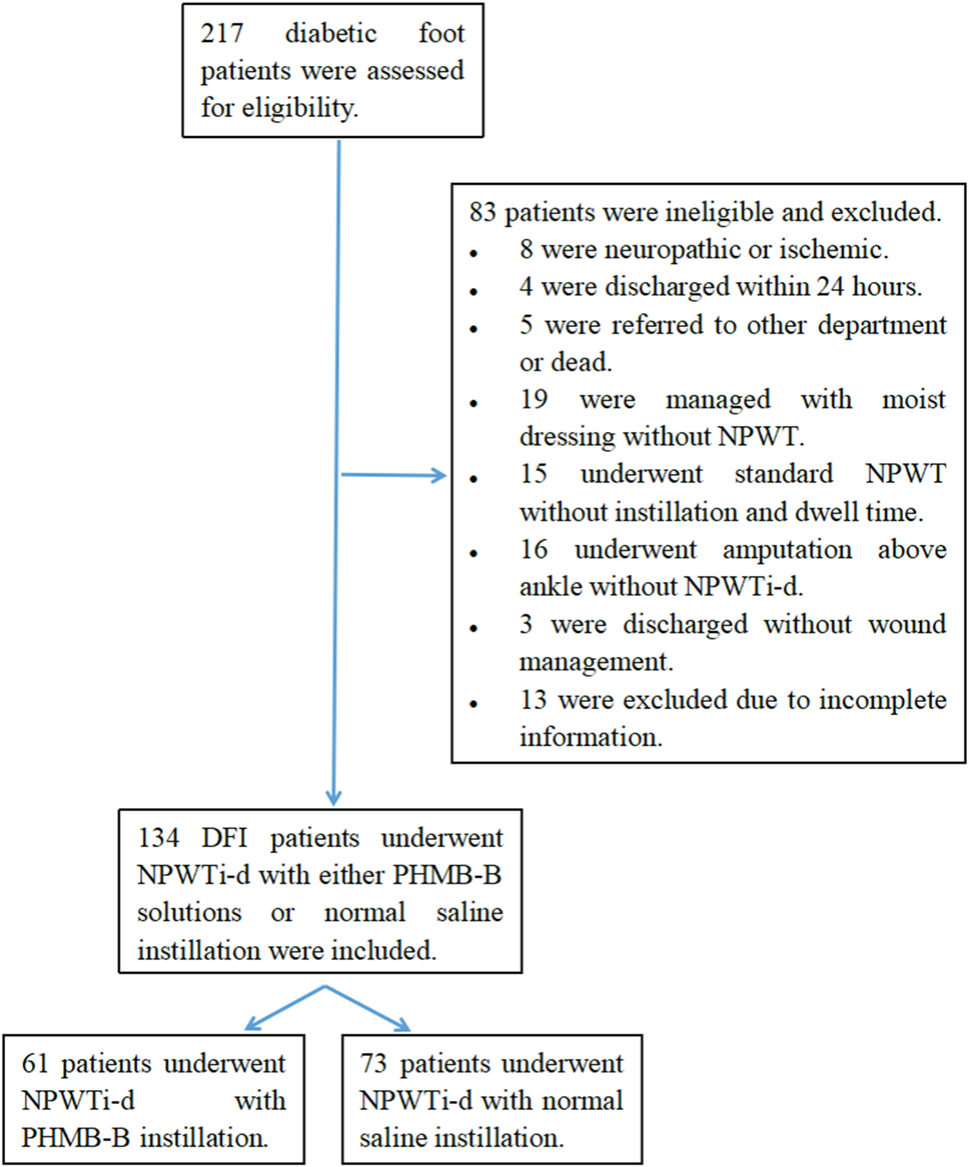
Flowchart of enrollment of study participants. DFI, diabetic foot infection; NPWT, negative pressure wound therapy; NPWTi-d, negative pressure wound therapy with instillation and dwell time; PHMB-B, 0.1% polyhexamethylene and 0.1% biguanide.

A total of 134 consecutive patients with DFIs were included in the analysis, with 73 receiving NS as an irrigation solution for NPWTi-d and 61 receiving PHMB-B instillation for NPWTi-d during the aforementioned time period (Fig. 1). Age, sex, body mass index, current smoking status, and medical comorbidities were comparable and did not show statistically significant differences between the two groups, as shown in Table 1.

**Table 1:** Demographic characteristics and baseline laboratory results on admission of diabetic foot infection patients.

Variable	Polyhexamethylene biguanide instillation (n = 61)	Normal saline instillation (n = 73)	*P* value
Sex, n (%)			
Male	49 (80.33%)	52 (71.23%)	0.2236
Female	12 (19.67%)	21 (28.77%)	
Age (years)	57.8 ± 12.9	59.9 ± 12.3	0.3316
Blood glucose (mmol/L)	12.9 ± 7.1	10.5 ± 5.3	0.0231
Glycosylated hemoglobin (HbA1c, %)	9.4 ± 2.0	9.7 ± 2.2	0.5471
C-reactive protein (mg/L)	81.3 ± 87.2	96.1 ± 73.2	0.4531
White blood cell (*10^9^/L)	11.6 ± 5.9	12.2 ± 6.1	0.5708
Neutrophil (%)	0.76 ± 0.72	0.75 ± 0.11	0.5632
Albumin (g/L)	31.5 ± 9.6	30.0 ± 6.3	0.2805
Body Mass Index (kg/m^2^)	23.8 ± 3.4	24.0 ± 3.7	0.7912
Procalcitonin (ng/mL)	1.8 ± 4.1	1.1 ± 2.3	0.4941

Additionally, the laboratory test results upon admission of both groups indicated no significant statistical difference, with the exception of a higher percentage of fasting glucose in the PHMB-B NPWTi-d group compared to the NS NPWTi-d group (*p* = 0.0231) (Table 1).

The findings indicate that there is no statistically significant difference between the group receiving PHMB-B instillation and the group receiving NS irrigation in various outcomes: (1) length of hospital stay (31.7 ± 17.5 days *vs.* 33.3 ± 21.7 days; *p* = 0.6783); (2) duration of wound bed preparation (15.5 ± 8.6 days *vs.* 17.5 ± 13.3 days, *p* = 0.5034); (3) NPWTi-d application times (1.9 ± 0.9 times *vs.* 2.4 ± 1.5 times,* p* = 0.1458); (4)duration of intravenous antimicrobial administration (14.1 ± 12.9 days *vs.* 16.1 ± 12.4 days, *p* = 0.3567); (5) overall cost of hospitalization (89,935 ± 62,245 CNY *vs.* 95,266 ± 73,282 CNY, *p* = 6173) ; (6) and complication of NPWTi-d. Two patients (3.3%) in the PHMB-B group and four patients (5.5%) in the NS group experienced complications, specifically skin maceration. However, the disparity in complication rates between the two groups did not reach statistical significance (*p* = 0.6882).

## Discussion

In individuals with diabetes mellitus, foot infections may exhibit prolonged healing times compared to non-diabetic individuals, largely attributed to bacterial biofilm presence within the wound environment.

The evolution of NPWT technology has led to the development of NPWT with instillation and dwell time (NPWTi-d), which involves the application of topical solutions directly onto the wound bed followed by negative pressure removal. This method has shown to enhance wound cleansing, granulation tissue formation, and overall healing in non-responsive wounds compared to traditional NPWT techniques^26,27^.

The selection of an appropriate instillation solution is a topic of ongoing debate in the treatment of NPWTi-d. The ideal solution for topical instillation should possess both effective wound bioburden reduction properties and minimal local cytotoxicity^28^. Various solutions have been utilized for instillation in NPWTi-d, with the literature predominantly focusing on active antibacterial agents such as sodium hypochlorite (Dakin's solution), silver nitrate, acetic acid, polyhexanide, povidone-iodine, bacitracin, and sulfur-based solutions^29–35^. However, some studies have shown favorable results when using NS as an instillation solution in infected wounds, despite its lack of antimicrobial properties^27,28,36,37^.

Previous research in a porcine skin explant biofilm model has indicated the potential superiority of PHMB over NS in decreasing colony-forming units^38^. However, despite positive outcomes for both treatments, no significant clinical difference was observed between PHMB-B and NS as topical wound irrigation solution in the management of DFIs. This disparity in research methodologies may be attributed to the distinction between our human study and their ex vivo study. Furthermore, several studies comparing the efficacy of NS and PHMB-B in patients with infected wounds found no significant differences in rates of dehiscence, wound closure, wound recurrence, amputation, or mortality between the two solutions^39–41^. These findings are consistent with the results of our study. The clinical efficacy, cost-effectiveness, greater accessibility, non-irritating and non-allergenic properties of NS may position it as the preferred choice for expanding the utilization of NPWTi-d in larger patient populations^40,41^.

In this study, a custom-made NPWTi-d system was utilized in place of commercially available reticulated open-cell foam dressings specifically designed for use with NPWTi-d (V.A.C. VeraFlo Therapy, KCI, an Acelity company, San Antonio, TX), as a result of the unavailability of commercial products at our hospital. It is possible that the use of V.A.C. VeraFlo Therapy could yield different results, given that the NPWTi-d system incorporates topical fluid instillation, programmed dwell time, and a unique foam-wound interface that collectively offer potential benefits to patients with complex wounds^42^.

Data was collected from two different commercial NPWT devices utilized for NPWTi-d in the current research. Utilizing a single product is expected to mitigate bias and enhance the study's reliability. However, due to the retrospective nature of the study, the device type utilized cannot be altered. A retrospective study compared the efficacy of two NPWT systems (V.A.C. therapy, KCI, Inc., San Antonio, Texas *vs.* RENASYS NPWT system, Smith & Nephew, Hull, United Kingdom) in treating wounds of various causes. The research findings indicated that there were no significant clinical differences in effectiveness and functionality between the two predominant NPWT devices^43^.

However, it is important to acknowledge the limitations of this study, such as its retrospective nature and reliance on a single medical center, which hindered the ability to conduct proper randomization of study groups over an extended duration. Additionally, the lack of standardized wounds for comparison and the presence of patient heterogeneity further complicate the interpretation of the results. Given that a historical case series does not meet the criteria of an optimal matched pair or comparative design, our study was restricted to investigating the impact of two topical solutions of NPWTi-d for wound bed preparation before definitive closure, without delving into additional outcomes such as skin graft take rate or suture dehiscence. Moreover, different application systems and handmade components were utilized in the studies. It is important to note that our study primarily focused on the effects of NPWTi-d and did not include a comparison between Renasys-GOTM therapy and V.A.C.® Therapy System. Furthermore, there is a lack of long-term follow-up to evaluate outcomes of these two NPWTi-d solutions. Despite these limitations, our case series demonstrates promising results and contributes valuable insights into the selection of instillation solutions for NPWTi-d in the management of DFIs.

## Conclusions

The findings of this study indicate that NPWTi-d is an effective and safe adjunct therapy for DFIs treatment. Nevertheless, the use of NPWTi-d with PHMB-B as a topical instillation does not exhibit superiority over NS in the management of DFIs. Given the limitations of this study, it is recommended that future research endeavors, such as a robust randomized controlled trial designed prospectively with a large sample size, be conducted to further elucidate the efficacy of NPWTi-d with PHMB-B as a topical instillation in the treatment of DFIs.

## Data Availability

The datasets used and/or analyzed during the current study are available from the corresponding author on reasonable request.

## Abbreviations

NPWTi-d: Negative pressure wound therapy with instillation and dwell time
DFIs: Diabetic foot infections
PHMB-B: Polyhexamethylene biguanide and betaine
NS: Normal saline
DFU: Diabetic foot ulcer
PAD: Peripheral arterial disease
NPWT: Negative pressure wound therapy
